# Theoretical Tolerance Modelling of Dynamics and Stability for Axially Functionally Graded (AFG) Beams

**DOI:** 10.3390/ma16052096

**Published:** 2023-03-04

**Authors:** Jarosław Jędrysiak

**Affiliations:** Department of Structural Mechanics, Łódź University of Technology, al. Politechniki 6, 90-924 Łódź, Poland; jarek@p.lodz.pl

**Keywords:** axially functionally graded microstructured beams, tolerance-periodic microstructure, effect of microstructure, tolerance modelling, free vibrations

## Abstract

Some considerations of slender elastic nonperiodic beams are shown in this paper. These beams have a functionally graded structure on the macro-level along the *x*-axis, and a nonperiodic structure on the micro-level. The effect of the size of the microstructure on the behavior of the beams can play a crucial role. This effect can be taken into account by applying the tolerance modelling method. This method leads to model equations with slowly varying coefficients, some of which depend on the microstructure size. In the framework of this model, formulas of higher order vibration frequencies related to the microstructure can be determined, not only for the fundamental lower-order vibration frequencies. Here, the application of the tolerance modelling method was mainly shown to derive the model equations of the so-called general (extended) tolerance model and standard tolerance model, describing dynamics and stability for axially functionally graded beams with the microstructure. A simple example of free vibrations of such a beam was presented as an application of these models. The formulas of the frequencies were determined using the Ritz method.

## 1. Introduction

**Figure 1:**
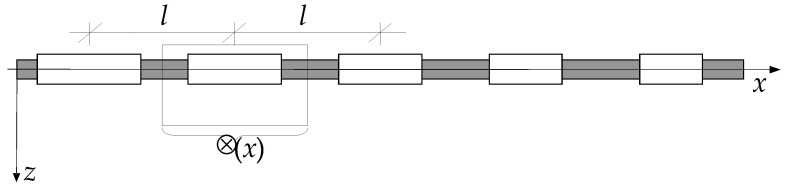
A part of a slender axially functionally graded beam with tolerance-periodic (nonperiodic) microstructure.

Slender axially functionally graded (AFG) beams with a tolerance-periodic (nonperiodic) microstructure are the main considered objects. These beams are made of many small elements, called cells, cf. Figure 1. Since a microstructure of beams is nonperiodic (tolerance-periodic) along their axis *x*, a macrostructure of them can be treated as functionally graded along the axis, cf. Suresh and Mortensen [1], Woźniak et al. [2]. Such microstructured beams can be widely applied in various branches of engineering.

Dynamics and stability problems of the considered AFG beams can be determined by a partial differential equation with noncontinuous, tolerance-periodic, highly oscillating coefficients. Because the governing equation in this form is not a good tool to analyze special problems, various approximated averaging methods were proposed.

For the modelling of thermomechanical problems of functionally graded structures with microheterogeneity, various averaging methods were adopted, which were also applied for periodic structures, cf. [1,2]. Many of these approaches lead to averaged models with so-called averaged (effective) properties of the structure (here: of the beam), Models that use the asymptotic homogenization can be mentioned, cf. Bensoussan et al. [3]; other models are applied to analyze plates with a periodic structure, cf. Kohn and Vogelius [4], while still others are applied to microperiodic beams, as Kolpakov demonstrated [5,6,7]. Unfortunately, the model governing equations usually neglect the effect of the microstructure size.

Different other modelling methods used to analyze various composite media were also proposed. Some issues concerning the effect of the microstructure on the properties of ceramic porous composites were analyzed, among others, in [8,9,10].

Periodic plates were also described using a homogenization with microlocal parameters, cf. [11]. The relationship between the 3D and the homogenization approach for the Euler–Bernoulli beams was presented in [12]. The problems of buckling for thin-walled columns made of multicells and having rectangular cross-sections were considered (theoretically, numerically and experimentally) in [13]. In paper [14], a dynamic critical load for buckling of columns was analyzed.

Vibrations of composite beams were considered using certain comprehensive research in [15]. Applying a modified couple stress theory and a meshless method, a model of bending with microstructure effects of laminated beams was formulated in [16]. A normal and layer-wise third-order shear deformable shell/plate theory was used to consider finite deformations of curved laminated beams with geometric nonlinearities in [17]. Numerical and/or mathematical modelling of bending or vibrations of composite beams were presented in [18,19,20]. Using analytical relations and finite element method, combined analytical-numerical models were proposed for composite auxetic beams—a torsion, cf. [21], or vibrations, cf. [22]. Numerical investigations of composite layered beams based on Carrera’s unified formulation were shown in [23].

For composite plates and shells, also with a functionally graded structure, there were formulated various models in many works. The strong formulation isogeometric analysis of composite plates was proposed in paper [24]. Both layer theory and differential quadrature method combined together were considered in [25]. The stability of annular three-layered plates made of fiber-reinforced facings and foam core was analyzed in [26]. The problems of stability for columns having closed cross-sections made of fiber-multilayered plate were analyzed in [27,28] using a semi-analytical method based on the laminate plate theory. The dynamics of composite auxetic plates were considered in [29]. The vibrations of porous functionally graded material plate resting on Winkler’s elastic foundation were considered in [30] applying the first-order shear deformation theory, combined with the variational approach and the Galerkin Vlasov’s method. The dynamic stiffness method was used to describe vibrations of functionally graded plates in [31]. The vibrations of nanocomposite functionally graded shells were modeled using a generalized differential quadrature method in some papers [32,33,34].

Thermomechanics problems of functionally graded beams were analyzed in a series of papers. Some free frequencies of sandwich beams having functionally graded composite core were considered using meshless methods in [35]. The effect of the shear correction function in modal analysis of composite beams with functionally graded structure was investigated in [36]. A one-dimensional theory with a generalization of layer-wise displacement approaches was proposed in [37] for dynamical problems of composite beams. In [38], free vibrations of composite nanobeams with functionally graded structure were optimized. The bending and/or vibrations of axially functionally graded composite beams were considered in [39,40,41]. The bending problems of composite beams with porosity having a functionally graded structure were analyzed in [42] using Eringen’s Nonlocal Theory.

However, the model equations obtained in the framework of the proposed modelling approaches for microstructured media usually neglect the effect of the microstructure size. On the other hand, this effect can be of great importance in vibrations of these media, cf. Brillouin [43], where the relations of micro- and macrovibrations with the micro- and macrostructure, respectively, were observed. In order to describe some similar problems, certain special methods were adopted to take into account this effect, which was considered and investigated for periodic structures in some works, e.g.,: in [44], the differential quadrature method was applied to analyze the vibration gaps for beams with a periodic structure; waves propagating in a periodic beam on a foundation were presented in [45]; waves propagating in beams with periodic stiffness were analysed using a multireflection method in [46]; free vibration frequencies of periodic beams were considered in [47]; in [48], vibration gaps of beams with a periodic structure were analysed using a transfer matrix method.

Different thermomechanical issues of microstructured (periodic and tolerance-periodic) media can be analyzed using an alternative approach, called *the tolerance modelling method* (or *the tolerance method*), cf. Woźniak and Wierzbicki [49], Woźniak et al. (eds) [2], [50]. This approach is helpful to apply in various problems, determined by differential equations having highly oscillating, noncontinuous functional coefficients. In the modelling procedure of this approach, these exact governing equations are replaced by the averaged equations of the model, having coefficients that are constant (or slowly varying functions). Some of these coefficients depend clearly on the size of the microstructure.

This method was applied to different problems of various periodic structures in many papers. The vibrations of periodic plane structures were investigated in [51]. An analysis of dynamics of one-directional periodic plates, with the length of the periodic cell greater than thickness, was shown in [52]. The dynamics of wavy-type periodic plates were presented in [53]. In the work [54], the vibrations of thin plates with periodic stiffeners were analyzed. The dynamics of periodic medium thickness plates were considered in [55]. The stability problems of periodic thin plates resting on a periodic subsoil were analyzed in [56]. A description of the dynamics for periodic thin plates having the thickness of an order microstructure size was proposed in [57]. The stability of periodic shells was analyzed in [58,59]. The dynamic problems of periodic medium thickness plates on a periodic subsoil were shown in [60]. Periodic thin plates with geometrical nonlinearities were analyzed in [61]. The vibrations for periodic slender geometrically nonlinear beams were considered in [62]. In [63], a certain tolerance model of dynamics for three-layered plate-type periodic structures was presented. A new modelling approach was proposed in [64] for stability and dynamics problems of slender visco-elastic beams with a periodic structure on a damped foundation. In [65], the distributions of stresses in thin composite plates with a periodic structure were investigated in multiscale. Mathematical modelling of thermoelasticity issues of cylindrical thin shells with microperiodic structure in two directions was presented in [66,67]. Stability analysis of slender periodic beams on elastic foundation was considered in [68].

Functionally graded structures with nonperiodic microheterogeneity were also successfully modeled applying the tolerance method. Heat conduction in transversally graded media was considered in [69]. The vibrations for longitudinally graded plates were analyzed in [70,71] and the buckling problems of such plates in [72]. Tolerance models of dynamics for thin-walled structures with a dense arrangement of ribs were applied in [73,74]. Heat transfer in cylindrical composite conductors having nonuniformly distributed constituents was described in [75,76]. Thermoelasticity problems for transversally graded laminates with the effect of the microstructure were analyzed in [77]. In [78], the free vibrations of thin functionally graded plates with one-directional microstructure were considered. The free vibration problem of functionally graded plates with medium thickness was modeled in [79]. The dynamic problems of thin functionally graded shells with microstructure were shown in [80,81] and the dynamics and stability of them in [82]. Theoretical analysis of the buckling problems for thin functionally graded microstructured plates on a subsoil was proposed in [83]. It should be noted that the above publications do not cover all issues considered by the authors and the literature on the method of tolerance modelling for microstructured media is not complete.

In this work, the main goal is to propose two new tolerance models of slender axially functionally graded (AFG) beams having a tolerance-periodic (nonperiodic) microstructure with axial force—the first, which will be named the “*standard*” or “classic” *tolerance model* (by using the definition of a slowly varying function) and the second, which will be called the “*general tolerance model*” (by using the definition of a weakly slowly varying function, which was presented also for beams with a periodic structure in [64]). The effect of the size of the microstructure on the mechanical behavior of the functionally graded beams with the microstructure under consideration is described by both derived models equations. This microstructural effect of these beams can be of great importance in problems of dynamics, for instance in free or forced vibrations. The tolerance models make it possible to analyze not only the fundamental lower vibrations (and frequencies), which are related to the beam structure in the macroscale, but also the higher order vibrations (and frequencies), cf. Brillouin [43], which are related to the beam structure in the microscale. Moreover, to evaluate results of both tolerance models, the governing equations of the asymptotic model are derived using the asymptotic homogenization procedure, which removes this microstructural effect. The proposed models are applied to a simple example of free vibrations for a functionally graded beam. The formulas of free vibration frequencies are obtained using the Ritz method. Hence, the originality/specificity of the study is to derive *two tolerance models of slender AFG beams having tolerance-periodic microstructure and with axial force*, and to apply them to a simple example to show the higher order free vibrations.

## 2. Preliminaries of Modelling

Introduce the Cartesian coordinate orthogonal system *Oxyz* and *t* as the time coordinate. The region of the undeformed beam is denoted by Ω≡{(x,y,z):−a/2≤y≤a/2, −h/2≤z≤h/2, x∈Λ}, with Λ as the beam axis, Λ≡[0,*L*]; *h*(·) as the beam height; *a*(·) as the beam width; *L* as the length of the beam. Let *∂* denote derivatives of *x*. Define the “basic cell” Δ≡[−*l*/2,*l*/2] on *Ox*, where parameter *l* is the length of cell Δ and satisfies the condition *h*_max_<<*l*<<*L*. Hence, parameter *l* can be called *the microstructure parameter*. It is assumed that the beam can have the cross-section dimensions as width *a*(·) and/or height *h*(·), being the tolerance-periodic (nonperiodic) functions in *x*, but the material properties as mass density *ρ* = *ρ*(·,*y*,*z*) and/or modulus of elasticity *E* = *E*(·,*y*,*z*) are tolerance-periodic (nonperiodic) functions in *x* and even functions in *y*, *z*. Denote by *w*(*x*,*t*) ($x\in \bar{\Delta }$) a beam deflection (along *z*-direction) and by *p*—all loadings along the *z*-direction.

Applying the known assumptions and relations of the theory for slender beams and denoting by:

(1) $$\begin{array}{ll} d(x)=\int_{-a/2}^{a/2}\int_{-h/2}^{h/2}E(x,y,z)z^{2}dzdy, & \\ \mu (x)=\int_{-a/2}^{a/2}\int_{-h/2}^{h/2}\rho (x,y,z)dzdy, & j(x)=\int_{-a/2}^{a/2}\int_{-h/2}^{h/2}\rho (x,y,z)z^{2}dzdy, \end{array}$$

tolerance-periodic functions in *x*: the bending stiffness of the beam *d*(·), the mass density μ(·); the rotational mass inertia *j*(·), the following governing equation of the axially tolerance-periodic (nonperiodic) beams (axially functionally graded beams) is obtained:

(2) $$\partial \partial (d\partial \partial w)-\partial (n\partial w)+\mu \ddot{w}-j\partial \partial \ddot{w}=p,$$

where *n* is the axial force.

Equation (2) has highly oscillating, noncontinuous, tolerance-periodic functional coefficients.

## 3. Tolerance Modelling Approach

### 3.1. Introductory Concepts

In the modelling, some concepts defined in [2,49,50] are used. These concepts are also presented and applied in various papers, e.g., in [83], but to make the paper self-consistent some of them are represented in the form adapted for the beams, cf. [64].

Denote a cell at *x*∈Λ_Δ_ as Δ(*x*) ≡ *x* + Δ, Λ_Δ_ = {*x*∈Λ: Δ(*x*)⸦Λ}. Define for an integrable function *f* the *averaging operator* as:

(3) $$<f>(x)=l^{-1}\int_{\Delta (x)}f(\xi )d\xi ,\;x\in \Lambda ,\;\xi \in \Delta (x).$$

For a periodic function *f*, the averaged value calculated from (3) is constant, but for the tolerance-periodic function, this value is the slowly varying function in *x*.

Let the *k*-th gradient of function f=f(x),  x∈Λ, be denoted by $\partial^{k}f$, k=0,1,…,α, (for problems of these beams α = 2); $\partial^{0}f\equiv f$; and ${\tilde{f}}^{\left(k\right)}(\cdot ,\cdot )$ be a function defined in $\bar{\Lambda }\times R^{m}$.

*The tolerance-periodic function* can be called function $f\in H^{2}(\Lambda )$, $f\in TP_{\delta }^{2}(\Lambda ,\Delta )$, if for k=0,1,2, the following conditions hold:

$$\begin{array}{l} (i)\;(\forall x\in \Lambda )\;(\exists {\tilde{f}}^{\left(k\right)}(x,\cdot )\in H^{0}(\Delta ))\;[||\partial^{k}f\left|{}_{\Lambda_{\Delta }}\right.(\cdot )-{\tilde{f}}^{\left(k\right)}(x,\cdot )|{|}_{H^{0}(\Lambda ,\Delta )}\leq \delta ],\\ (ii)\;\int_{\Delta (\cdot )}{\tilde{f}}^{\left(k\right)}(\cdot ,\xi )d\xi \in C^{0}(\bar{\Lambda }), \end{array}$$

where δ is *the tolerance parameter*, δ<<1, related to the problems under consideration; and function ${\tilde{f}}^{\left(k\right)}(x,\cdot )$ is the periodic approximation of $\partial^{k}f$ in Δ(x), x∈Λ, k=0,1,2.

Function $F\in H^{2}(\Lambda )$ is called *the weakly-slowly-varying function*, $F\in WSV_{\delta }^{2}(\Lambda ,\Delta )$, if

$$\begin{array}{l} (i)\;F\in TP_{\delta }^{2}(\Lambda ,\Delta ),\\ (ii)\;(\forall (x;\xi )\in \Lambda )\;[(x;\xi )\Rightarrow F(x)\approx F(\xi )\wedge \partial F(x)\approx \partial F(\xi )], \end{array}$$

Function $F\in H^{2}(\Lambda )$ is called *the slowly-varying function*, $F\in SV_{\delta }^{2}(\Lambda ,\Delta )$, if

$$\begin{array}{l} (i)\;F\in WSW_{\delta }^{2}(\Lambda ,\Delta ),\\ (ii)\;(\forall x\in \Lambda )\;l|\partial F(x)|{|}_{\Delta (x)}\approx 0. \end{array}$$

Function $\phi \in H^{2}(\Lambda )$ is called *the highly oscillating function*, $\phi \in HO_{\delta }^{2}(\Lambda ,\Delta )$, if

$$\begin{array}{l} (i)\;\phi \in TP_{\delta }^{2}(\Lambda ,\Delta ),\\ (ii)\;(\forall x\in \Lambda )\;[{\tilde{\phi }}^{\left(k\right)}(x,\cdot ){|}_{\Delta (x)}=\partial^{k}\tilde{\phi }(x),\;k=0,1,2],\\ (iii)\;\forall \;F\in SV_{\delta }^{2}(\Lambda ,\Delta )\;\exists f\equiv \phi F\in TP_{\delta }^{2}(\Lambda ,\Delta )\;\;{\tilde{f}}^{\left(k\right)}(x,\cdot ){|}_{{}_{\Delta (x)}}=F(x)\partial^{k}\tilde{\phi }(x){|}_{{}_{\Delta (x)}},\;k=1,2. \end{array}$$

For *k* = 0 let us denote $\tilde{f}\equiv {\tilde{f}}^{\left(0\right)}$.

Introduce a highly oscillating function *g*(·), defined on $\bar{\Lambda }$, $g\in HO_{\delta }^{2}(\Lambda ,\Delta )$, continuous together with gradient *∂*^1^*g*. Gradient *∂*^2^*g* is piecewise continuous and bounded. Function *g*(·) is *the fluctuation shape function* of the second kind, $FS_{\delta }^{2}(\Lambda ,\Delta )$, if it depends on *l* as a parameter and the conditions hold:

- *∂^k^g*∈*O*(*l*^α−*k*^) for *k* = 0,1,…,α, α = 2, *∂*^0^*g* ≡ *g*,
- <*g*>(*x*) ≈ 0 for every $x\in \Lambda_{\Delta }$,

with *l* as the microstructure parameter. It should be noted that the second of the above conditions can be replaced by <μ*g*>(*x*) ≈ 0 for every *x*∈Λ_Δ_, where μ > 0 is some tolerance-periodic function.

### 3.2. Assumptions of Modelling

The general assumptions of the tolerance modelling were formulated in [2,49,50] and were adopted to special problems of different structures in many papers using the introductory concepts. Here, these assumptions are presented for the considered beams.

*The micro–macro decomposition* is the first assumption, which is formulated for the deflection of the beam *w* in the form:

(4) $$w(x,t)=U(x,t)+g^{A}(x)Q^{A}(x,t),A=1,\ldots ,M,\;x\in \Lambda ,$$

where *U*(·,*t*) and *Q^A^*(·,*t*) are new kinematic unknown functions, named, respectively, *the macrodeflection* and *the fluctuation amplitudes*. They are assumed to be weakly-slowly-varying functions of the second kind, i.e., $U(\cdot ,t),\;Q^{A}(\cdot ,t)\in WSV_{\delta }^{2}(\Lambda ,\Delta )$, or to be slowly varying functions of the second kind, i.e., $U(\cdot ,t),\;Q^{A}(\cdot ,t)\in SV_{\delta }^{2}(\Lambda ,\Delta )$. Functions *g^A^*(·), *A* = 1,…,*M*, are assumed to be the known fluctuation shape functions, being postulated a priori in the task under consideration and determining the unknown fields (here: the deflection of the beam) oscillations related to a microstructure of the beam. These functions should meet the following constraints:

*∂^k^g^A^*∈*O*(*l*^2−*k*^) for *k* = 0,1,2,

<μ*g^A^*> = 0,

<μ*g^A^g^B^*> = 0 for *A* ≠ *B*, *A*, *B* = 1,…,*M*.

*The tolerance averaging approximation* is the second assumption, where components of the order *O*(δ) are assumed to be negligibly small, e.g., for $q\in TP_{\delta }^{2}(\Lambda ,\Delta ),$ $Q\in SV_{\delta }^{2}(\Lambda ,\Delta )$ or $Q\in WSV_{\delta }^{2}(\Lambda ,\Delta )$, $g^{A}\in FS_{\delta }^{2}(\Lambda ,\Delta ),$ in:

(5) $$\begin{array}{l} <q>(x)=<\bar{q}>(x)+O(\delta ),\\ <qQ>(x)=<q>(x)Q(x)+O(\delta ),\\ <q\partial (g^{A}Q)>(x)=<q\partial g^{A}>(x)Q(x)+O(\delta ). \end{array}$$

The third assumption is called *the axial force restriction*, in which it is assumed that the terms with fluctuating parts of the axial force, $\tilde{n}(\cdot )\in TP_{\delta }^{2}(\Lambda ,\Delta )$ can be omitted in comparison to terms with averaged parts, $N(\cdot )\in SV_{\delta }^{2}(\Lambda ,\Delta )$, i.e.:

(6) $$\begin{array}{ll} n(x)=N(x)+\tilde{n}(x), & \\ N=<n>, & <\tilde{n}>=0. \end{array}$$

### 3.3. Modelling Procedure

The tolerance modelling procedure can be applied as in [49], [2] or [50]. However, this one here is similar to the one shown in [2]. At the beginning of the tolerance modelling procedure, the micro–macro decomposition (4) is substituted into Equation (2). After it, the dynamic governing Equation (2) is not satisfied, i.e., within the macrodynamics a residual field *r*(·), is defined:

(7) $$r=\partial \partial (d\partial \partial (U+g^{A}Q^{A})-\partial (n\partial (U+g^{A}Q^{A}))+\mu (\ddot{U}+g^{A}{\ddot{Q}}^{A})-j\partial \partial (\ddot{U}+g^{A}{\ddot{Q}}^{A})-q.$$

Now, introducing *the residual orthogonality condition*, the following conditions are imposed on the residual field *r*(·):

(8) $$<r>(x,t)=0,\;\;\;<rg^{B}>(x,t)=0.$$

Using conditions (8), combined with the modelling assumptions of the tolerance averaging approximation (5) and the axial force restriction (6), a system of equations for the macrodeflection *U*(·,*t*) and the fluctuation amplitudes *Q^A^*(·,*t*), *A* = 1,…,*M*, can be derived. These equations depend on the specification of the class of slowly-varying functions *U*(·,*t*), *Q^A^*(·,*t*) (weakly-slowly-varying or slowly-varying function).

## 4. Asymptotic Modelling Approach

### 4.1. Basic Assumptions

Introduce some denotations: ε, ε∈(0,1], as a small parameter; Δ_ε_≡[−ε*l*/2, ε*l*/2] as an interval; Δ_ε_(*x*) ≡ *x* + Δ_ε_, $x\in \bar{\Lambda }$, as ε-cell; a function $\tilde{q}(x,\cdot )\in H^{1}(\Delta )$, ∀$x\in \bar{\Lambda }$; functions ${\tilde{q}}_{\varepsilon }(x,y)\equiv \tilde{q}(x,y/\varepsilon )$, where ${\tilde{q}}_{\varepsilon }(x,\cdot )\in H^{1}(\Delta_{\varepsilon })\subset H^{1}(\Delta ),$*y*∈Δ_ε_(x), $x\in \bar{\Lambda }$; and moreover independent functions *g^A^*(·), *g^A^*(·)∈*HO*_δ_, *A* = 1,…,*M*, and their periodic approximations ${\tilde{g}}^{A}(x,\cdot )$, given as ${\tilde{g}}_{\varepsilon }^{A}(x,y)\equiv {\tilde{g}}^{A}(x,y/\varepsilon )$, *y*∈Δ_ε_(*x*), $x\in \bar{\Lambda }$.

The fundamental assumption of the asymptotic modelling is *the asymptotic decomposition* for the beam deflection *w*(*x*,*t*), which can be written in the form:

(9) $$w_{\varepsilon }(x,y,t)=U(y,t)+\varepsilon^{2}{\tilde{g}}_{\varepsilon }^{A}(x,y)Q^{A}(y,t),$$

where *y*∈Δ_ε_(*x*), *t*∈(*t*_0_,*t*_1_), and functions *w*, *U*, *Q^A^* (*A* = 1,…,*M*) are continuous and bounded in $\bar{\Lambda }$ with their first derivatives. Denoting:

(10) $$\hat{\partial }{\tilde{g}}_{\varepsilon }^{A}(x,y)\equiv \varepsilon \partial {\tilde{g}}^{A}{\left.\left(x,\bar{y}\right)\right|}_{\bar{y}=y/\varepsilon },\;\;\;\hat{\partial }\hat{\partial }{\tilde{g}}_{\varepsilon }^{A}(x,y)\equiv \partial \partial {\tilde{g}}^{A}{\left.\left(x,\bar{y}\right)\right|}_{\bar{y}=y/\varepsilon },$$

some selected derivatives of the beam deflection *u*_ε_ take the form:

(11) $$\begin{array}{l} \partial w_{\varepsilon }(x,y,t)=\partial U(y,t)+\hat{\partial }{\tilde{g}}_{\varepsilon }^{A}(x,y)Q^{A}(y,t)+\varepsilon^{2}{\tilde{g}}_{\varepsilon }^{A}(x,y)\partial Q^{A}(y,t),\\ \partial \partial w_{\varepsilon }(x,y,t)=\partial \partial U(y,t)+\hat{\partial }\hat{\partial }{\tilde{g}}_{\varepsilon }^{A}(x,y)Q^{A}(y,t)+\\ \;\;\;\;+2\hat{\partial }{\tilde{g}}_{\varepsilon }^{A}(x,y)\partial Q^{A}(y,t)+\varepsilon^{2}{\tilde{g}}_{\varepsilon }^{A}(x,y)\partial \partial Q^{A}(y,t),\\ \partial {\dot{w}}_{\varepsilon }(x,y,t)=\partial \dot{U}(y,t)+\hat{\partial }{\tilde{g}}_{\varepsilon }^{A}(x,y){\dot{Q}}^{A}(y,t)+\varepsilon^{2}{\tilde{g}}_{\varepsilon }^{A}(x,y)\partial {\dot{Q}}^{A}(y,t),\\ {\dot{w}}_{\varepsilon }(x,y,t)=\dot{U}(y,t)+\varepsilon^{2}{\tilde{g}}_{\varepsilon }^{A}(x,y){\dot{Q}}^{A}(y,t). \end{array}$$

Applying the limit passage ε→0 for Formulas (9), (11) the expressions are obtained:

(12) $$\begin{array}{l} w_{\varepsilon }(x,y,t)=U(x,t)+O(\varepsilon ),\\ \partial w_{\varepsilon }(x,y,t)=\partial U(x,t)+O(\varepsilon ),\\ \partial \partial w_{\varepsilon }(x,y,t)=\partial \partial U(x,t)+\hat{\partial }\hat{\partial }{\tilde{g}}_{\varepsilon }^{A}(x,y)Q^{A}(x,t)+O(\varepsilon ),\\ {\dot{w}}_{\varepsilon }(x,y,t)=\dot{U}(x,t)+O(\varepsilon ),\;\;\;\partial {\dot{w}}_{\varepsilon }(x,y,t)=\partial \dot{U}(x,t)+O(\varepsilon ). \end{array}$$

Components of an order ε (or higher), *O*(ε), in Equation (12) can be omitted with the parameter ε going to zero.

### 4.2. Modelling Procedure

For the asymptotic model, Lagrange’s function $L_{\varepsilon }=L(x,y/\varepsilon ,\partial \partial U,\partial U,U,Q^{A},\partial \dot{U},\dot{U})$, *y*∈Δ_ε_(*x*), $x\in \bar{\Lambda }$, *t*∈(*t*_0_,*t*_1_), can be introduced in the form:

(13) $$\begin{array}{l} L_{\varepsilon }=\frac{1}{2}<\mu {\dot{w}}_{\varepsilon }{\dot{w}}_{\varepsilon }+j\partial {\dot{w}}_{\varepsilon }\partial {\dot{w}}_{\varepsilon }>-\frac{1}{2}<d\partial \partial w_{\varepsilon }\partial \partial w_{\varepsilon }+n\partial w_{\varepsilon }\partial w_{\varepsilon }>+<pw_{\varepsilon }>=\\ =\frac{1}{2}(<\mu >\dot{U}\dot{U}+<j>\partial \dot{U}\partial \dot{U})+<p>U-\\ -\frac{1}{2}(<d>\partial \partial U\partial \partial U+2<d\hat{\partial }\hat{\partial }{\tilde{g}}_{\varepsilon }^{A}>\partial \partial UQ^{A}+<d\hat{\partial }\hat{\partial }{\tilde{g}}_{\varepsilon }^{A}\hat{\partial }\hat{\partial }{\tilde{g}}_{\varepsilon }^{B}>Q^{A}Q^{B}+N\partial U\partial U). \end{array}$$

Using the asymptotic modelling, for ε→0 function *L*_ε_ of variable *y*/ε, *y*∈Δ_ε_(*x*), tends to an averaged Lagrange’s function *L*_0_, taking the form:

(14) $$\begin{array}{l} L_{0}=\frac{1}{2}(<\mu >\dot{U}\dot{U}+<j>\partial \dot{U}\partial \dot{U})+<p>U-\\ -\frac{1}{2}(<d>\partial \partial U\partial \partial U+2<d\partial \partial g^{A}>\partial \partial UQ^{A}+<d\partial \partial g^{A}\partial \partial g^{B}>Q^{A}Q^{B}+N\partial U\partial U). \end{array}$$

Applying to Lagrange’s function (14) the averaged asymptotic Euler–Lagrange equations, the governing equations of the asymptotic model for the considered beams can be obtained.

## 5. Model Equations

### 5.1. General Tolerance Model Equations

Using the *residual orthogonality condition* (8) with the tolerance modelling assumptions (5), (6), the following averaged coefficients are introduced:

(15) $$\begin{array}{ccc} D=<d>, & D^{A}=<d\partial \partial g^{A}>, & D^{AB}=<d\partial \partial g^{A}\partial \partial g^{B}>,\\ \tilde{m}<\mu >, & {\tilde{m}}^{A\;}=<{\mu g}^{A}>l^{-2}, & {\tilde{m}}^{AB}=<{\mu g}^{A}g^{B}>l^{-4},\\ \vartheta =<j>, & \vartheta^{AB}=<j\partial g^{A}\partial g^{B}>l^{-2},\\ N=<n>, & N^{AB}=<n\partial \partial g^{A}g^{B}>l^{-2}, & {\overset{⌣}{N}}^{AB}=-<n\partial g^{A}\partial g^{B}>l^{-2}=-N^{AB},\\ P=<p>, & P^{A}=<pg^{A}>l^{-2};\\ {\bar{N}}^{A}=<ng^{A}>l^{-2}, & {\bar{N}}^{AB}=<ng^{A}g^{B}>l^{-4}\overset{}{,}\;\\ {\bar{D}}^{A}=<dg^{A}>l^{-2}, & {\bar{D}}^{AB}=<dg^{A}g^{B}>l^{-4},\;\\ {\hat{D}}^{AB}=<d\partial g^{A}\partial g^{B}>l^{-2}, & {\overset{⌣}{D}}^{AB}=<d\partial \partial g^{A}g^{B}>l^{-2},\;\\ {\bar{\vartheta }}^{A}=<jg^{A}>l^{-2}, & {\bar{\vartheta }}^{AB}=<jg^{A}g^{B}>l^{-4}; \end{array}$$

The averaged equations of the axially functionally graded (axially tolerance-periodic) beams for *U*(·,*t*) and *Q^A^*(·,*t*) are formulated:

(16) $$\begin{array}{c} \partial \partial [D\partial \partial U+D^{A}Q^{A}+\underline{l^{2}\partial \partial \left({\bar{D}}^{A}Q^{A}\right)}]-\\ -\partial \left(N\partial U\right)+\tilde{m}\ddot{U}-\partial (\vartheta \partial \ddot{U})+l^{2}{\tilde{m}}^{A}{\ddot{Q}}^{A}-\underline{l^{2}\partial ({\bar{N}}^{A}\partial Q^{A}+{\bar{\vartheta }}^{A}{\ddot{Q}}^{A})}=P,\\ D^{A}\partial \partial U+D^{AB}Q^{B}+l^{2}{\tilde{m}}^{A}\ddot{U}+l^{2}\left(l^{2}m^{AB}+\vartheta^{AB}\right){\ddot{Q}}^{B}-l^{2}N^{AB}Q^{B}\overset{}{+}\\ +\underline{l^{2}\partial \partial \left({\bar{D}}^{A}\partial \partial U+l^{2}{\bar{D}}^{AB}\partial \partial Q^{B}\right)}+\underline{l^{2}[2({\overset{⏝}{D}}^{AB}-2{\hat{D}}^{AB})-l^{2}{\bar{N}}^{AB}]\partial \partial Q^{B}}-\\ -\underline{l^{2}\partial \left({\bar{\vartheta }}^{A}\partial \ddot{U}+l^{2}{\bar{\vartheta }}^{AB}\partial {\ddot{Q}}^{B}\right)}=l^{2}P^{A}. \end{array}$$

Equation (16) is the governing model equation of *the general tolerance model of slender elastic axially functionally graded beams with tolerance-periodic microstructure*. Coefficients of the above equations are slowly varying functions in *x*. Because some of those terms involve the microstructure parameter *l*, these equations make it possible to analyse the effect of the size of the microstructure on the stability and/or dynamic behaviour of the considered beams. The additional components to *the standard tolerance model* equations have been underlined. Equation (16) represents the system of *M*+1 differential equations for the new basic kinematic unknowns: the macrodeflection *U* and the fluctuation amplitudes *Q^A^*, *A* = 1,…,*M*, that must be *weakly-slowly-varying functions* in *x*. It can be observed that two boundary conditions should be formulated for each unknown (*U* and *Q^A^*, *A* = 1,…,*M*) at both ends of the beam (four conditions for each unknown).

### 5.2. Standard Tolerance Model Equations

After applying the condition (8) to the field (7) together with the tolerance modelling assumptions (5), (6) and the concept of the slowly varying function, averaged coefficients such as (15) can be denoted:

(17) $$\begin{array}{lll} D=<d>, & D^{A}=<d\partial \partial g^{A}>, & D^{AB}=<d\partial \partial g^{A}\partial \partial g^{B}>,\\ \tilde{m}=<\mu >, & {\tilde{m}}^{A}=<\mu g^{A}>l^{-2}, & {\tilde{m}}^{AB}=<\mu g^{A}g^{B}>l^{-4},\;\\ \vartheta =<j>, & \vartheta^{AB}=<j\partial g^{A}\partial g^{B}>l^{-2},\\ N=<n>, & N^{AB}=<n\partial \partial g^{A}g^{B}>l^{-2}, & {\overset{⌣}{N}}^{AB}=-<n\partial g^{A}\partial g^{B}>l^{-2}=-N^{AB},\\ P=<p>, & P^{A}=<pg^{A}>l^{-2}; \end{array}$$

The following model equations for *U*(·,*t*) and *Q^A^*(·,*t*) can be written:

(18) $$\begin{array}{l} \partial \partial (D\partial \partial U+D^{A}Q^{A})-\\ -\partial (N\partial U)+\tilde{m}\ddot{U}-\partial (\vartheta \partial \ddot{U})+l^{2}{\tilde{m}}^{A}{\ddot{Q}}^{A}=P,\\ D^{A}\partial \partial U+D^{AB}Q^{B}+l^{2}{\tilde{m}}^{A}\ddot{U}+l^{2}(l^{2}m^{AB}+\vartheta^{AB}){\ddot{Q}}^{B}-l^{2}N^{AB}Q^{B}\overset{}{=}l^{2}P^{A}. \end{array}$$

Equation (18) represents the governing equation of *the standard tolerance model of elastic slender axially functionally graded beams with tolerance-periodic microstructure*. These equations have coefficients being slowly varying functions in *x*. They describe the effect of the microstructure size on the overall stability and dynamic behavior of the considered beams in terms of the microstructure parameter *l*. Equation (18) represents the system of *M* + 1 differential equations for basic new unknowns: the macrodeflection *U* and *the fluctuation amplitudes Q^A^*, *A* = 1,…,*M*, which have to be *slowly-varying functions* in *x*. At both ends of the beam, two boundary conditions should be formulated only for the macrodeflection *U* (a total of four conditions), but not for the unknowns *Q^A^*, *A* = 1,…,*M*.

### 5.3. Equations of Asymptotic Model

The results calculated in the framework of the above models applying the tolerance modelling approach can be evaluated by introducing the approximate model, defined by the governing differential equations that remove the effect of the size of the microstructure. These equations can be obtained using the asymptotic modelling procedure, cf. [2], and are here adapted (Section 4) to the differential equation of the considered beams. Thus, applying the averaged asymptotic Euler–Lagrange equations together with asymptotic approximations of Lagrange’s function (14), and selected denotations (17), the model equations have the form:

(19) $$\begin{array}{l} \partial \partial (D\partial \partial W+D^{A}Q^{A})-\partial (N\partial W)+\tilde{m}\ddot{W}-\partial (\vartheta \partial \ddot{W})=P,\\ D^{A}\partial \partial W+D^{AB}Q^{B}=0\overset{}{.} \end{array}$$

Equation (19) represents *the asymptotic model of elastic slender axially functionally graded beams with tolerance-periodic microstructure*, neglecting the effect of the size of the microstructure on the mechanical behaviour of the beams. The above equations have coefficients that are slowly-varying functions in *x*, such as Equations (16) or (18) for both tolerance models, and unlike Equation (2), whose coefficients are noncontinuous, highly oscillating, tolerance-periodic and functional. At both ends of the beam, two boundary conditions have to be formulated only for the macrodeflection *U* (a total of four conditions). By the way, it can also be noted that Equation (19) can be derived directly from Equation (18) after the vanishing components with the microstructure parameter *l*.

## 6. Free Vibrations of a Special AFG Beam

### 6.1. Introduction

**Figure 2:**
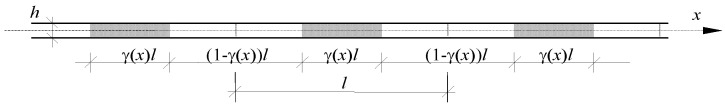
Fragment of axially functionally graded (AFG) beam with tolerance-periodically distributed mass density.

An elastic axially functionally graded (AFG) beam, being simply supported at the ends, having the length *L*, without the influence of the rotational inertia and the axial force, is an object of consideration. The microstructure of the beam is related to the tolerance-periodic mass density distribution (Figure 2).

**Figure 3:**
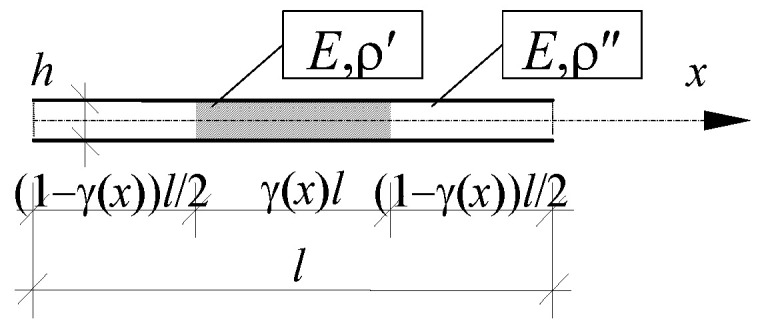
“Basic cell” of axially functionally graded beam under consideration.

The material property: Young’s modulus *E*; the dimensions of the beam cross section: height *h* and width *a*, are assumed to be constant. Load *p* is omitted. The tolerance-periodic distribution of mass density is assumed in the form:

(20) $$\rho (\cdot ,y)=\left\{\begin{array}{l} \rho \prime \;\;,\;\mathrm{for}\;y\in ((1-\gamma (x))l/2,(1+\gamma (x))l/2),\\ \rho \prime \prime ,\;\mathrm{for}\;y\in [0,(1-\gamma (x))l/2]\cup [(1+\gamma (x))l/2,l], \end{array}\right.$$

where γ(*x*) is a distribution function of material properties, cf. Figure 3.

In our considerations, only one fluctuation shape function is assumed, i.e., *g* = *g*^1^, *A* = *M* = 1, (thus, denote also *Q* = *Q*^1^). The form of the fluctuation shape function *g* should be related to the structure of the “basic cell” shown in Figure 3. Thus, the periodic approximation of the fluctuation shape function can be assumed as:

(21) $$\tilde{g}(x,\xi )=l^{2}[\cos(2\pi \xi /l)+c(x)],\xi \in \Delta (x),\;x\in \Lambda ,$$

where parameter *c*(*x*) is a slowly-varying function in *x*, determined by $<\tilde{\mu }\tilde{g}>=0$:

(22) $$c=c(x)=\{\sin[\pi \tilde{\gamma }(x)](\rho \prime -\rho \prime \prime )\}{\left\{\pi \{\rho \prime \tilde{\gamma }(x)+\rho \prime \prime [1-\tilde{\gamma }(x)]\}\right\}}^{-1},$$

where $\tilde{\gamma }(x)$ is the periodic approximation of the distribution function of material properties γ(*x*). Parameter *c*(*x*) is treated as constant in calculations of derivatives $\partial \tilde{g},\;\partial \partial \tilde{g}$.

The averaged coefficients (15) different from zero have the form:

(23) $$\begin{array}{ll} d=\frac{1}{12}Eah^{3},\\ D=d, & D^{11}=d<\partial \partial g\partial \partial g>,\\ \tilde{m}=<\mu >, & {\tilde{m}}^{11}=l^{-4}<\mu gg>;\\ {\bar{D}}^{1}=l^{-2}d<g>, & {\bar{D}}^{11}=l^{-4}d<gg>,\\ {\hat{D}}^{11}=l^{-2}d<\partial g\partial g>, & {\overset{⌣}{D}}^{11}=l^{-2}d<\partial \partial gg>. \end{array}$$

### 6.2. Free Vibration Equations

The governing equations of free vibration for the AFG beam with the tolerance-periodic microstructure introduced above will be determined within the aforementioned averaged models—the general and standard tolerance models, and the asymptotic model.

- The general tolerance model

The general tolerance model Equations (16) for the considered beam, with the averaged coefficients (23), can be written as:

(24) $$\begin{array}{l} D\partial \partial \partial \partial U+\tilde{m}\ddot{U}+\underline{l^{2}{\bar{D}}^{1}\partial \partial \partial \partial Q}=0,\\ \underline{l^{2}{\bar{D}}^{1}\partial \partial \partial \partial U}+D^{11}Q+l^{4}{\tilde{m}}^{11}\ddot{Q}+\underline{l^{4}{\bar{D}}^{11}\partial \partial \partial \partial Q}+\underline{2l^{2}({\overset{⌣}{D}}^{11}-2{\hat{D}}^{11})\partial \partial Q}=0. \end{array}$$

The underlined terms in Equations (24) are additional in the comparing to the standard tolerance model equations. The above equations describe both—*macrovibrations* (related to the averaged macrostructure of the considered beam) and *microvibrations* (related to the microstructure of the beam).

- The standard tolerance model

The standard tolerance model governing Equations (18) for the beam under consideration, with averaged coefficients (23) takes the form:

(25) $$\begin{array}{l} D\partial \partial \partial \partial U+\tilde{m}\ddot{U}=0,\\ D^{11}Q+l^{4}{\tilde{m}}^{11}\ddot{Q}=0. \end{array}$$

Similar to Equations (24) and (25), they describe also both the *macrovibrations* and the *microvibrations*.

- The asymptotic model

The asymptotic model governing Equations (19) for the considered beam, using coefficients (23), take the form:

(26) $$(a)\;D\partial \partial \partial \partial U+\tilde{m}\ddot{U}=0,\;\;\;(b)\;D^{11}Q=0.$$

It can be noticed that only one differential equation of the AFG beam with the tolerance-periodic microstructure is obtained in contrast to the tolerance models—general and standard. Only the macrovibrations of the beam are described by Equation (26a)_1_. Thus, the effect of the size of the beam microstructure, manifested, for example, in higher order vibrations, is omitted.

It should be noted that coefficients of the model Equations (24)–(26) are slowly varying functions in *x*.

### 6.3. Free Vibration Frequencies—The Ritz Method Applied to the Model Equations

Formulas for free vibration frequencies of the simply supported AFG beam with the tolerance-periodic microstructure assumed above will be determined and the calculational results will be presented.

It is too difficult to find analytical solutions of Equations (24)–(26), because they have slowly varying, functional coefficients. Hence, approximate formulas of free vibration frequencies can be derived using the known Ritz method, cf. [2,50]. In order to obtain these formulas, relations of the maximal energies—strain $E_{\max}$ and kinetic $K_{\max}$, have to be determined.

Solutions to Equations (24)–(26) should be assumed in the form satisfying boundary conditions for the simply supported beam:

(27) $$U(x,t)=A_{U}\sin(kx)\cos(\omega t),\;\;Q(x,t)=A_{Q}\sin(kx)\cos(\omega t),$$

where *k* is a wave number, ω is a free vibrations frequency. Introduce the denotations:

(28) $$\begin{array}{c} \overset{⌣}{B}=\int_{0}^{L}D\sin^{2}(kx)dx=\frac{{ah}^{3}}{12}E\int_{0}^{L}[\sin({kx)]}^{2}dx,\overset{⏜}{B}=\int_{0}^{L}D^{1}\sin^{2}\left(kx\right)dx=0,\\ \bar{B}=\int_{0}^{L}D^{11}\sin^{2}\left(kx\right)dx=\frac{a{\left(\pi h\right)}^{3}}{3}2\pi E\int_{0}^{L}[\sin({kx)]}^{2}dx,\\ \tilde{D}=\int_{0}^{L}D^{1}\sin^{2}\left(kx\right)dx=\frac{{ah}^{3}}{12}E\int_{0}^{L}c(x)[\sin({kx)]}^{2}dx,\\ \bar{D}=\int_{0}^{L}{\bar{D}}^{11}\sin^{2}\left(kx\right)dx=\frac{ah^{3}}{24}E\int_{0}^{L}[\sin({kx)]}^{2}dx+\frac{ah^{3}}{12}E\int_{0}^{L}{[c(x)]}^{2}[\sin({kx)]}^{2}dx,\\ \overset{⌣}{D}=\int_{0}^{L}{\overset{⌣}{D}}^{11}\cos^{2}\left(kx\right)dx=\frac{{ah}^{3}}{12}\pi E\int_{0}^{L}c(x)[\cos({kx)]}^{2}dx,\\ \hat{D}=\int_{0}^{L}{\hat{D}}^{11}\cos^{2}\left(kx\right)dx=\frac{\pi^{2}ah^{3}}{6}E\int_{0}^{L}[\cos({kx)]}^{2}dx\\ \overset{⌣}{\mu }=\int_{0}^{L}\tilde{m}\sin^{2}(kx)dx=ah\int_{0}^{L}\{[1-\tilde{\gamma }(x)]\rho \prime \prime +\tilde{\gamma }(x)\rho \prime \}[\sin({kx)]}^{2}dx,\\ \bar{\mu }=\int_{0}^{L}{\tilde{m}}^{11}\sin^{2}\left(kx\right)dx=\frac{ah}{4\pi }\int_{0}^{L}\{(\rho \prime -\rho \prime \prime )[2\pi \tilde{\gamma }(x)+\sin(2\pi \tilde{\gamma }(x))]+2\pi \rho \prime \prime \}[\sin({kx)]}^{2}dx+\\ \;\;+\frac{ah}{\pi }\left(\rho \prime -\rho \prime \prime \right)\int_{0}^{L}c\left(x\right)[\pi c\left(x\right)\tilde{\gamma }(x)-2\sin(\pi \tilde{\gamma }(x))][\sin({kx)]}^{2}dx+\\ \;\;+ah\rho \prime \prime \int_{0}^{L}{[c(x)}^{2}[\sin({kx)]}^{2}dx, \end{array}$$

and by applying (27) the formulas of the maximal energies—strain $E_{\max}$ and kinetic $K_{\max}$—the framework of the three considered models can be derived:

- the general tolerance model

(29) $$\begin{array}{l} E_{\max}^{GT}=\frac{1}{2}\{A_{U}{}^{2}k^{4}\overset{⌣}{B}+2l^{2}A_{U}k^{4}A_{Q}\tilde{D}+l^{4}A_{Q}{}^{2}k^{4}\bar{D}-2l^{2}A_{Q}{}^{2}k^{2}[\overset{⌣}{D}-2\hat{D}]+A_{Q}{}^{2}\bar{B}\},\\ K_{\max}^{GT}=\frac{1}{2}\omega^{2}\{A_{U}{}^{2}\overset{⌣}{\mu }+l^{4}A_{Q}{}^{2}\bar{\mu }\}; \end{array}$$

- the standard tolerance model

(30) $$\begin{array}{l} E_{\max}^{ST}=\frac{1}{2}\{A_{U}{}^{2}k^{4}\overset{⌣}{B}+A_{Q}{}^{2}\bar{B}\},\\ K_{\max}^{ST}=\frac{1}{2}\omega^{2}\{A_{U}{}^{2}\overset{⌣}{\mu }+l^{4}A_{Q}{}^{2}\bar{\mu }\}; \end{array}$$

- the asymptotic model

(31) $$\begin{array}{l} E_{\max}^{AM}=\frac{1}{2}\{A_{U}{}^{2}k^{4}\overset{⌣}{B}+A_{Q}{}^{2}\bar{B}\},\\ K_{\max}^{AM}=\frac{1}{2}\omega^{2}\{A_{U}{}^{2}\overset{⌣}{\mu }\}. \end{array}$$

The conditions of the Ritz method have the form:

(32) $$\frac{\partial (E_{\max}-K_{\max})}{\partial A_{U}}=0,\frac{\partial (E_{\max}-K_{\max})}{\partial A_{Q}}=0.$$

By applying the conditions (32) to Formulas (29)–(31), the following characteristic equations can be written for all considered models:

- the general tolerance model

(33) $$\begin{array}{l} \omega^{4}l^{4}\overset{⌣}{\mu }\bar{\mu }-\omega^{2}\{\overset{⌣}{\mu }[l^{4}k^{4}\bar{D}-2l^{2}k^{2}(\overset{⌣}{D}-2\hat{D})+\bar{B}]+l^{4}k^{4}\bar{\mu }\overset{⌣}{B}\}+\\ +k^{4}\overset{⌣}{B}[l^{4}k^{4}\bar{D}-2l^{2}k^{2}(\overset{⌣}{D}-2\hat{D})+\bar{B}]-{\left(l^{2}k^{4}\tilde{D}\right)}^{2}=0; \end{array}$$

- the standard tolerance model

(34) $$\omega^{4}l^{4}\overset{⌣}{\mu }\bar{\mu }-\omega^{2}(\overset{⌣}{\mu }\bar{B}+l^{4}\bar{\mu }k^{4}\overset{⌣}{B})+k^{4}\overset{⌣}{B}\bar{B}=0;$$

- the asymptotic model

(35) $$\omega^{2}\overset{⌣}{\mu }-k^{4}\overset{⌣}{B}.$$

By solving the above equations, the formulas of the free vibration frequencies for the three considered models can be obtained.

Hence, from Equation (33), the formulas of *the free vibration frequencies for the general tolerance model* take the form:

(36) $$\omega_{-,+}=\sqrt{\begin{array}{l} \frac{\overset{⌣}{\mu }[\bar{B}+l^{4}k^{4}\bar{D}-2l^{2}k^{2}(\overset{⌣}{D}-2\hat{D})]+l^{4}k^{4}\bar{\mu }\overset{⌣}{B}}{2l^{4}\overset{⌣}{\mu }\bar{\mu }}\mp \\ \mp \frac{\sqrt{{\left\{l^{4}k^{4}\bar{\mu }\overset{⌣}{B}-\overset{⌣}{\mu }[\bar{B}-2l^{2}k^{2}(\overset{⌣}{D}-2\hat{D})+l^{4}k^{4}\bar{D}]\right\}}^{2}+{\left(2l^{4}k^{4}\tilde{D}\right)}^{2}\overset{⌣}{\mu }\bar{\mu }}}{2l^{4}\overset{⌣}{\mu }\bar{\mu }} \end{array}},$$

where: ω_−_ is *the fundamental lower free vibration frequency* related to the averaged structure of the considered AFG beam in the macroscale; ω_+_ is *the higher free vibration frequency* related to the beam structure in the microscale.

Solving Equation (34), the formulas of *the free vibration frequencies for the standard tolerance model* are calculated:

(37) $$(a)\;{\tilde{\omega }}_{-}=\sqrt{\frac{\overset{⌣}{B}k^{4}}{\overset{⌣}{\mu }}},\;\;(b)\;{\tilde{\omega }}_{+}=\sqrt{\frac{\bar{B}}{l^{4}\bar{\mu }}},$$

where: ${\tilde{\omega }}_{-}$ is *the fundamental lower free vibration frequency* related to the averaged structure of the AFG beam in the macroscale; ${\tilde{\omega }}_{+}$ is *the higher free vibration frequency* related to the beam structure in the microscale.

However, from Equation (35), only one formula of *the free vibration frequency for the asymptotic model* is obtained:

(38) $$\omega_{0}=\sqrt{\frac{\overset{⌣}{B}k^{4}}{\overset{⌣}{\mu }}},$$

which is *the fundamental lower free vibration frequency* related to the averaged structure of the AFG beam in the macroscale.

It can be observed that for the considered example of the AFG beam, the formulas of fundamental lower frequency in the framework of the standard tolerance model, (37a)_1_, and the asymptotic model, (38), are identical.

### 6.4. Free Vibrations—Calculating Results

Calculations are made for the following distribution functions of material properties γ(*x*):

(39a) $$\tilde{\gamma }(x)=\sin^{2}(\pi x/L),$$

(39b) $$\tilde{\gamma }(x)=\cos^{2}(\pi x/L),$$

(39c) $$\tilde{\gamma }(x)={\left(x/L\right)}^{2},$$

(39d) $$\tilde{\gamma }(x)=\sin(\pi x/L),$$

(39e) $$\tilde{\gamma }(x)=0.5,$$

where Formula (39e)_5_ determines an example of a periodic beam.

Introduce the dimensionless frequency parameters:

(40) $$(a)\;\Omega \equiv l\sqrt{\frac{\rho \prime }{E}}{\tilde{\omega }}_{-}=l\sqrt{\frac{\rho \prime }{E}}\omega_{0},\;\;(b)\;\Omega_{+}^{\mathrm{ST}}\equiv l\sqrt{\frac{\rho \prime }{E}}{\tilde{\omega }}_{+},\;\;(c)\;\Omega_{-}^{\mathrm{GT}}\equiv l\sqrt{\frac{\rho \prime }{E}}\omega_{-},\;\;(d)\;\Omega_{+}^{\mathrm{GT}}\equiv l\sqrt{\frac{\rho \prime }{E}}\omega_{+},$$

with the free vibration frequencies $\omega_{0}={\tilde{\omega }}_{-}$, ${\tilde{\omega }}_{+}$, ω_−_ and ω_+_ defined by Equations (36) and (37), respectively.

**Figure 4:**
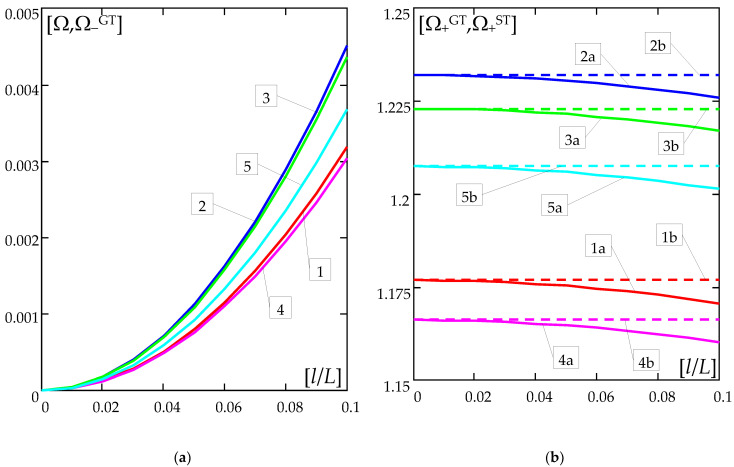
Dimensionless frequency parameters (40) versus nondimensional microstructure parameter (*l*/*L*) (for *h*/*l* = 0.1): (**a**) lower frequency parameters $\Omega ,\Omega_{-}^{\mathrm{GT}}$ (for *ρ*″/*ρ*′ = 0.2), (**b**) higher frequency parameters $\Omega_{+}^{\mathrm{ST}},\Omega_{+}^{\mathrm{GT}}$ (for *ρ*″/*ρ*′ = 0.8); (symbols of curves: 1—γ by (39a)_1_; 2—γ by (39b)_2_; 3—γ by (39c)_3_; 4—γ by (39d)_4_; 5—γ by (39e)_5_; a—by the general tolerance model (GTM), b—by the standard tolerance model (STM)).

**Figure 5:**
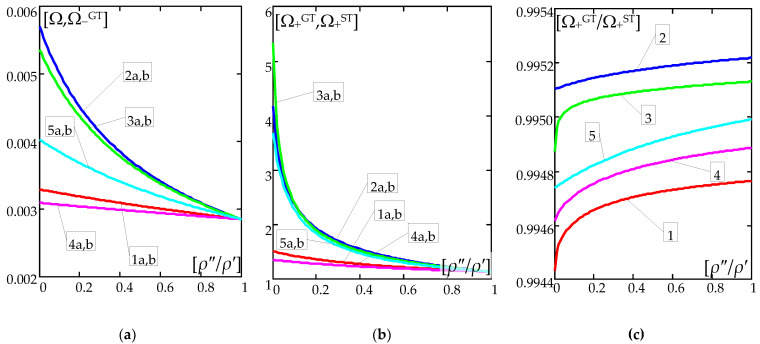
Dimensionless frequency parameters (40a), (40c)_1,3_ versus mass density ratio (*ρ*″/*ρ*′) (for *h*/*l* = 0.1, *l*/*L* = 0.1) for: (**a**) lower frequency parameters $\Omega ,\Omega_{-}^{\mathrm{GT}}$; (**b**) higher frequency parameters $\Omega_{+}^{\mathrm{ST}},\Omega_{+}^{\mathrm{GT}}$; (**c**) ratios of higher frequency parameters $\Omega_{+}^{\mathrm{GT}}/\Omega_{+}^{\mathrm{ST}}$; (symbols of curves: 1—γ by (39a)_1_; 2—γ by (39b)_2_; 3—γ by (39c)_3_; 4—γ by (39d)_4_; 5—γ by (39e)_5_; a—by the general tolerance model (GTM), b—by the standard tolerance model (STM)).

The numerical results are shown in Figure 4 and Figure 5. Figure 4 presents the plots of the frequency parameters versus the nondimensional microstructure parameter *l*/*L*. Figure 5 presents the curves of the frequency parameters versus ratio *ρ*″/*ρ*′. Figure 4a and Figure 5a show the plots of the lower frequency parameters $\Omega ,\Omega_{-}^{\mathrm{GT}}$, but Figure 4b and Figure 5b show the plots of higher frequency parameters $\Omega_{+}^{\mathrm{ST}},\Omega_{+}^{\mathrm{GT}}$. Diagrams of ratios $\Omega_{+}^{\mathrm{GT}}/\Omega_{+}^{\mathrm{ST}}$ are shown in Figure 5c. All these results are calculated for: ratio *h*/*l* = 0.1, parameter *k* = π; and for the various distribution functions of material properties γ(*x*), (39). Moreover, Figure 4a is made for *ρ*″/*ρ*′ = 0.2, Figure 4b for *ρ*″/*ρ*′ = 0.8; and Figure 5 for ratio *l*/*L* = 0.1. In Figure 4 and Figure 5, the curves denoted by numbers “1–5” present the results calculated for the distribution functions γ of the material properties, cf. (39): 1—γ by (39a)_1_; 2—γ by (39b)_2_; 3—γ by (39c)_3_; 4—γ by (39d)_4_; 5—γ by (39e)_5_. The letters “a-b”, on the other hand, denote the results obtained using the appropriate model: a—the general tolerance model, b—the standard tolerance model.

The effect of the different distributions of the mass density is shown in Figure 4 and Figure 5.

It can be observed that for the considered distribution functions of mass density (39), the highest values of the lower frequency parameters are obtained for all ratios *ρ*″/*ρ*′∈[0;1] for function γ(*x*) by (39b)_2_, then the smaller values of these parameters are in order for functions γ(*x*) given by: (39c)_3_, (39e)_5_, (39a)_1_, (39d)_4_., cf. Figure 4a and Figure 5a.

A similar descending order is for the values of the higher frequency parameters, i.e., respectively, for the function γ(*x*) according to (39b)_2_, by (39c)_3_, (39e)_5_, (39a)_1_, to (39d)_4_, but for the range *ρ*″/*ρ*′∈(0.1,1]. However, for *ρ*″/*ρ*′ ≤ 0.1, the decreasing values of these frequency parameters are, respectively, for the functions: (39c)_3_, (39b)_2_, (39e)_5_, (39a)_1_, (39d)_4_.

For the considered case of the beam formulas, the lower free vibration frequencies in the framework of the standard tolerance and the asymptotic models are identical, cf. (37a)_1_, (38). However, values of the lower frequency parameters by both the tolerance models—general and standard—are nearly identical, cf. Figure 4a and Figure 5a.

It may be noticed that the higher frequency parameters by the general tolerance model are smaller than these parameters by the standard tolerance model. Moreover, the higher frequency parameters by the standard tolerance model are independent of the nondimensional microstructure parameter *l*/*L*, but these parameters by the general model depend on this ratio *l*/*L*, cf. Figure 4b.

However, the differences between the higher frequency parameters calculated using the general and standard tolerance models are very small, cf. Figure 5b,c.

## 7. Final Remarks

The vibrations and stability of slender axially functionally graded beams with tolerance-periodic (nonperiodic) microstructure have been considered here. Applying *the tolerance modelling method,* the known differential equation of slender beams has been averaged. This equation with noncontinuous, tolerance-periodic (nonperiodic) coefficients has been replaced by the system of governing differential equations with continuous, smooth, slowly-varying coefficients.

The tolerance modelling leads to two various tolerance models, respectively, *the general tolerance model* and *the standard tolerance model*, using two different functions—*the weakly-slowly-varying function* and *the slowly-varying function*. The derived tolerance model equations take into account the effect of the microstructure size on the overall dynamic and stability behaviour of the beams under consideration. Hence, both tolerance models enable the analysis of this effect in these problems at both the macro- and micro-levels.

In contrast to the standard tolerance model equations, the general tolerance model equations of the axially functionally graded slender beams with the tolerance-periodic microstructure involve additional components, which make it necessary to take into account the effect of the size of the beam microstructure also in stationary issues.

In order to evaluate the obtained calculation results, the differential equations of *the asymptotic model* have also been derived by applying the asymptotic modelling procedure. It should be noted that this model makes it possible to analyse vibrations and stability problems at the macro-level only, neglecting the effect of the size of the beam microstructure.

From the results presented in the considered simple example, it seems that both the proposed tolerance models—general and standard—for the axially functionally graded slender beams with the microstructure can be good tools for investigating the effect of the size of the beam microstructure on the vibrations of these beams.

The governing equations of the general and standard tolerance models of the considered beams will be used to investigate other, more interesting and complicated problems of the dynamics or/and stability in forthcoming works.
